# The ratio of non-high-density lipoprotein cholesterol to high-density lipoprotein cholesterol is associated with diabetic kidney disease: A cross-sectional study

**DOI:** 10.1371/journal.pone.0311620

**Published:** 2024-11-27

**Authors:** Liling Zhang, Di Fan, Tingting Zhu, Lei Geng, Linwang Gan, Santao Ou, Defeng Yin

**Affiliations:** 1 Department of Nephrology, The Affiliated Hospital, Southwest Medical University, Sichuan Province, P. R. China; 2 Department of Anesthesiology, The Affiliated Hospital, Southwest Medical University, Sichuan Province, P. R. China; 3 Department of Emergency, The Affiliated Hospital, Southwest Medical University, Sichuan Province, P. R. China; Neyshabur University of Medical Sciences, ISLAMIC REPUBLIC OF IRAN

## Abstract

Non-High-Density Lipoprotein Cholesterol to High-Density Lipoprotein Cholesterol Ratio (NHHR) is a significant indicator of atherosclerosis. However, its association with diabetic kidney disease (DKD) remains unclear. This study aims to explore the relationship between NHHR and the prevalence of DKD among the U.S. adults using data from the National Health and Nutrition Examination Survey (NHANES) spanning 1999 to 2020. Participants were selected based on the stringent inclusion and exclusion criteria. We utilized single-factor analysis, multivariate logistic regression, and smooth curve fitting to investigate the relationship between NHHR and DKD. Our study included 8,329 diabetic individuals, who were categorized into DKD and non-DKD groups based on the presence or absence of kidney damage. A significant difference in NHHR was observed between these groups. After adjusting for potential confounders, we found that NHHR was positively associated with the prevalence of DKD. Specifically, each one-unit increase in NHHR corresponded to a 6% rise in the prevalence of DKD, with this association remaining significant across stratified NHHR values. Threshold effect analysis revealed an inflection point at an NHHR of 1.75, beyond this point, each unit increase in NHHR was associated with a 7% increase in the prevalence of DKD. Subgroup analysis confirmed the robustness of these findings. Our study demonstrates a significant correlation between NHHR and DKD prevalence, suggesting that monitoring NHHR could be an effective strategy for reducing DKD prevalence.

## Introduction

Diabetes is a prevalent endocrine disorder characterized by elevated blood glucose levels, primarily resulting from pancreatic beta-cell dysfunction and subsequent insulin deficiency [1,2]. The incidence of diabetes is high, with its complications impacting multiple organs. As of 2019, diabetes affected approximately 460 million people globally, making it the eighth leading cause of mortality worldwide [3]. By 2021, the number of patients with diabetes had risen to 529 million, reflecting a prevalence of 6.1%, and an increasing trend. Projections suggest that by 2050, the global diabetic population will double [4].

The rise in diabetes prevalence has been accompanied by an increase in DKD, a major complication affecting 30%-40% of individuals with diabetes. DKD is a significant contributor to end-stage renal disease (ESRD), and is associated with elevated mortality, posing a substantial challenge to global public health [5]. Therefore, early identification and prevention of DKD is crucial to mitigating this challenge.

Currently, the primary biomarkers for diagnosing DKD in renal structure and function are the estimated glomerular filtration rate (eGFR) and the urine albumin to creatinine ratio (UACR). Although these indicators are used to predict mortality in DKD, their association with DKD prevalence is not well understood [6,7]. DKD pathogenesis is complex, involving disturbances in lipid metabolism and oxidative stress. Current treatments that focus on lowering blood glucose and urinary protein levels do not fundamentally alter DKD progression [8–10]. Abnormalities in cholesterol metabolism have been shown significantly impact DKD with disrupted lipid metabolism contributing to excessive reactive oxygen species, and worsening renal injury [11].

High-density lipoprotein cholesterol (HDL-C) is known for its protective role in cardiovascular diseases (CVD) by reducing oxidative stress and promoting anti-apoptotic effects [12,13]. Non-HDL-C, which includes low-density lipoprotein cholesterol (LDL-C), intermediate-density lipoprotein, and very-low-density lipoprotein cholesterol (VLDL-C), has garnered attention for its role in CVD. Evidence suggests that non-HDL-C exacerbates diabetes and atherosclerosis, making it a critical factor to monitor in patients with cardiovascular disease [14,15]. Insulin resistance in diabetes, leads to increased production of VLDL particles and small dense LDL (sd-LDL) particles, which, when oxidized, enhance vascular permeability, macrophage aggregation, and atherosclerotic plaque formation, thus serving as a superior predictor of CVD [16–19]. Non-High-Density Lipoprotein Cholesterol to High-Density Lipoprotein Cholesterol Ratio (NHHR) integrates the positive role of HDL-C and the negative role of non-HDL-C, offering enhanced predictive value and diagnostic efficiency for lipid-related diseases compared to traditional indicators. NHHR, has proven valuable in assessing and predicting conditions such as diabetes, depression, stroke, periodontitis, and abdominal aortic aneurysm [20–24]. However, the relationship between NHHR and DKD remains largely unexplored. This study hypothesize a correlation between NHHR and DKD based on the premise that lipid metabolism disorders influence DKD progression. Utilizing NHANES data from 1999 to 2020, this research aims to provide a novel perspective for the advanced detection and diagnosis of DKD.

## Materials and methods

### Study population

**Fig 1:**
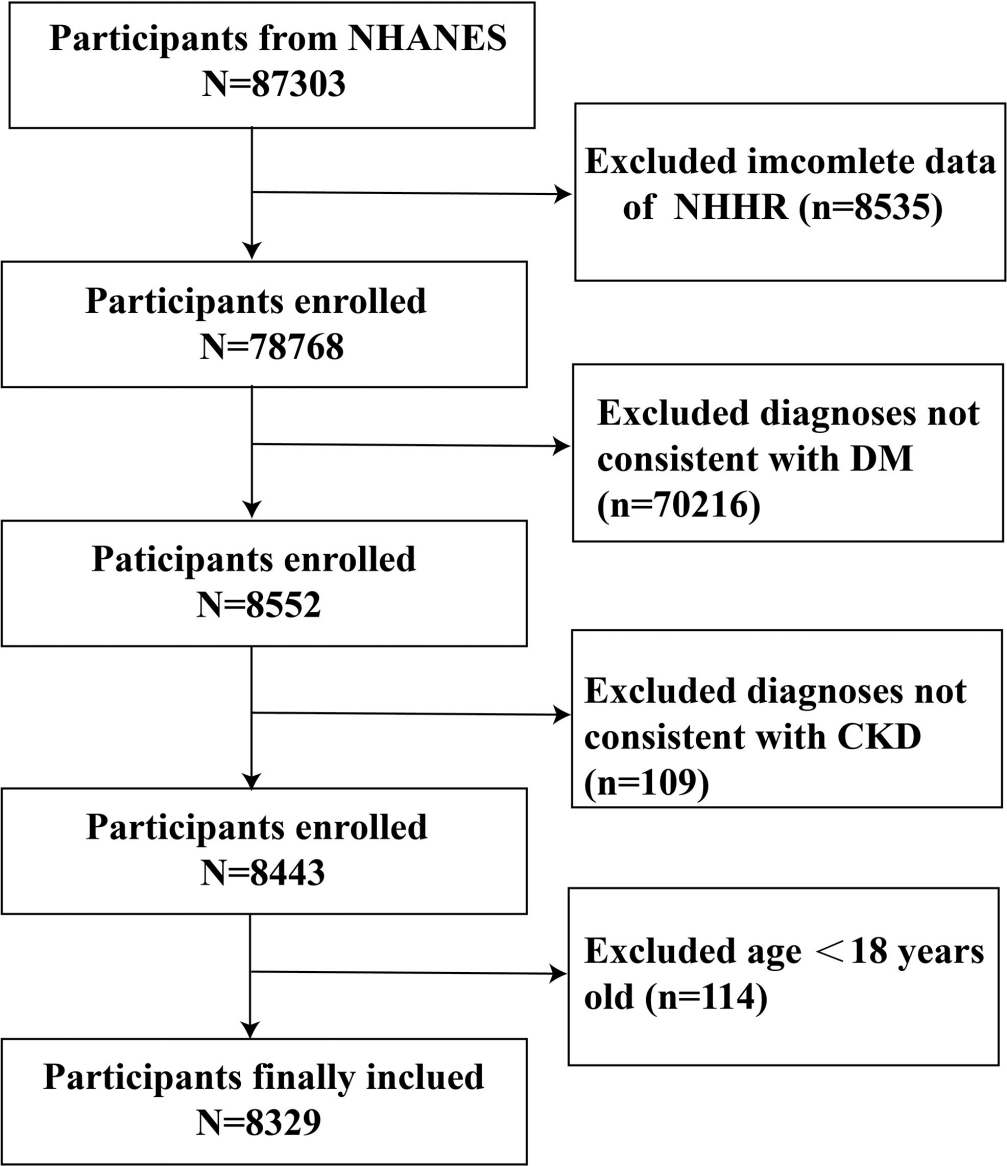
The flowchart of enrolled participants. DM, diabetes mellitus.

Data for this study were obtained from the National Health and Nutrition Examination Survey (NHANES), a nationally representative database that focuses on nutrition and health risk factors. Data collection was conducted through face-to-face interviews and mobile medical examination centers [25]. We utilized data from 1999 to 2020. After applying strict inclusion and exclusion criteria, 8,329 diabetic participants were included in the analysis. A flowchart detailing participant inclusion and exclusion is presented (Fig 1).

### Exposure and outcome

NHHR represents the ratio of cholesterol composition, which was the primary exposure variable. NHHR is calculated as total cholesterol (TC) minus HDL-C, which is non-high-density lipoprotein cholesterol (non-HDL-C), and then divided by HDL-C [21]. Both TC and HDL-C were determined using enzymatic methods.


$$NHHR=\frac{TC-HDL\_C}{HDL\_C}$$

DKD served as the outcome variable. Diabetes was defined in four ways: (1) self-reported diagnosis of diabetes; (2) fasting blood glucose ≥ 7.0 mmol/L; (3) glycated hemoglobin ≥ 6.5%; or (4) current use of insulin or oral hypoglycemic medications. Participants meeting these criteria were further classified as having DKD if they also had a urine UACR ≥ 30 mg/g and or an estimated glomerular filtration rate (eGFR) < 60 mL/min/1.73 m^2^, were further redefined as DKD. eGFR was calculated using the CKD-EPI equation [26]: For men with serum creatinine (Scr) ≤ 0.9 mg/dL, eGFR = 141 × (Scr / 0.9) ^-0.411^ × (0.993) ^age^; for men with Scr > 0.9 mg/dL, eGFR = 141 × (Scr / 0.9) ^-1.209^ × (0.993) ^age^; for women with Scr ≤ 0.7 mg/dL, eGFR = 144 × (Scr / 0.7) ^-0.329^ × (0.993) ^age^; for women with Scr > 0.7 mg/dL, eGFR = 144 × (Scr / 0.7) ^-1.209^ × (0.993) ^age^. UACR (mg/g) = urine albumin (mg/dL) / urine creatinine (g/dL) [27]. The solid-phase fluorescence immunoassay (FIA) was used for quantifying urine albumin, while the enzyme method was used for determining fasting blood glucose levels, and the Jaffe rate method was employed to quantify creatinine.

### Covariates

Potential confounding variables were collected from NHANES questionnaires and included race, gender, age, marital status, education level, body mass index (BMI), poverty income ratio (PIR), and medical history. Medical history data included diagnoses of hypertension, stroke, cancer, coronary heart disease, hypercholesterolemia and liver disease. According to the response to the question “Have you smoked at least 100 cigarettes in your lifetime?”, participants answering “Yes” were further classified as “Former smoking” or “Currently smoking” based on the answer to the question “Do you currently smoke?”. Participants were finally categorized into “Currently smoking”, “Former smoking” or “Never smoking”. BMI was stratified into three categories: ≤ 25, 25–30, and > 30 kg/ m^2^. PIR was also stratified into three categories: ≤ 1, 1–3, and > 3.

### Statistical analysis

Diabetic individuals were categorized into DKD and non-DKD groups based on whether they met the criteria for DKD. Continuous variables were summarized as mean ± standard deviations, while categorical variables were reported as percentages. T-tests and chi-square tests were used to assess inter-group differences. Multivariate logistic regression analyses were conducted to examine the association between NHHR and DKD. The independent associations between NHHR and DKD were tested in three different models, with the continuous variable NHHR classified as categorical variables through quartiles, and the *p*-value for trend was used to assess the consistency of the relationship. Model 1 was adjusted for no covariates; Model 2 was adjusted for race, gender, and age; all potential confounding covariates were adjusted in Model 3. Furthermore, smooth curve fitting and threshold effect analysis were employed to explore the relationship between NHHR and DKD prevalence and to identify any inflection points. Subgroup analyses were performed to examine potential heterogeneity in the association between NHHR and DKD. R software (version 4.3) and Empower Stats 4.0 were used for statistical analyses.

### Ethics statement

The data used in this study were sourced from the publicly available NHANES database. All participants provided informed consent, and the study was approved by the national ethics committee.

## Results

### Baseline characteristic

Table 1 presents the characteristics of 8,329 participants, with a mean age of 61.09 ± 13.87 years. The cohort comprised 52.59% males and 47.41% females. Significant differences were observed between the DKD and the non-DKD groups in terms of age, race, marital status, education level, hypertension, hypercholesterolemia, coronary heart disease, stroke, cancer, smoking, PIR, and TC. The DKD group was characterized by older age, a higher proportion of smokers, and an increased prevalence of hypertension, hypercholesterolemia, stroke, and cancer, but had lower PIR, education levels, and TC (Table 1).

**Table 1:** Characteristics of the included participants.

Characteristic	Overall (8329)	Non-DKD (4677)	DKD (3652)	*P*-value
**Gender, n(%)**				0.073
**male**	4380 (52.59)	2419 (51.72)	1961 (53.70)	
**female**	3949 (47.41)	2258 (48.28)	1691 (46.30)	
**Age (year)**	61.09 ± 13.87	57.63 ± 13.48	65.54 ± 13.07	<0.001
**Race, n(%)**				<0.001
**Mexican American**	1669 (20.00)	999 (21.36)	670 (18.35)	
**Other Hispanic**	784 (9.40)	502 (10.73)	282 (7.72)	
**Non-Hispanic White**	2939 (35.29)	1600 (34.21)	1339 (36.66)	
**Non-Hispanic Black**	2154 (25.86)	1097 (23.46)	1057 (28.94)	
**Other Races**	783 (9.40)	479 (10.24)	304 (8.32)	
**Marriage, n(%)**				<0.001
**Married**	5318(63.85)	3140 (67.14)	2178 (59.64)	
**Widowed**	1057 (12.69)	406 (8.68)	651 (17.83)	
**Divorced**	812 (9.75)	439 (9.39)	373 (10.21)	
**Separated**	254 (3.05)	143 (3.06)	111 (3.04)	
**Never married**	628 (7.53)	382 (8.17)	246 (6.74)	
**Living with partner**	260 (3.12)	167 (3.57)	93 (2.55)	
**Education, n(%)**				<0.001
**Less than 9**^**th**^ **grade**	1619 (19.44)	847 (18.11)	772 (21.14)	
**9-11**^**th**^ **grade**	1436 (17.24)	745 (15.93)	691 (18.92)	
**High school graduate**	1909 (22.92)	1077 (23.03)	832 (22.78)	
**Some college/AA degree**	2172 (26.08)	1255 (26.83)	917 (25.11)	
**College graduate or above**	1193 (14.32)	753 (16.10)	440 (12.05)	
**Hypertension, n(%)**				<0.001
**Yes**	5408 (64.93)	2698 (57.69)	2710 (74.21)	
**No**	2921 (35.07)	1979 (42.31)	942 (25.79)	
**Hypercholesterolemia, n(%)**				0.004
**Yes**	5140 (61.71)	2823 (60.36)	2317 (63.44)	
**No**	3189 (38.29)	1854 (39.64)	1335 (36.56)	
**Coronary Heart Disease, n(%)**				<0.001
**Yes**	850 (10.00)	339 (7.25)	511 (13.99)	
**No**	7529 (90.00)	4338 (92.75)	3141 (86.01)	
**Stroke, n(%)**				<0.001
**Yes**	714 (8.60)	245 (5.24)	469 (12.84)	
**No**	7615 (91.4)	4432 (94.76)	3183 (87.16)	
**Liver Disease n(%)**				0.291
**Yes**	582 (7.00)	339 (7.25)	243 (6.65)	
**No**	7747 (93.00)	4338 (92.75)	3409 (93.35)	
**Cancer, n(%)**				<0.001
**Yes**	1107 (13.3)	539 (11.52)	568 (15.55)	
**No**	7222 (86.7)	4138 (88.48)	3084 (84.45)	
**Smoking**				<0.001
**Currently smoking, n(%)**	1366 (16.4)	809 (17.30)	557 (15.25)	
**Never smoking**	4159 (49.9)	2414 (51.61)	1745 (47.78)	
**Former smoking**	2804 (33.67)	1454 (31.09)	1350 (36.97)	
**PIR**	2.27± 1.45	2.37 ± 1.50	2.13 ± 1.37	<0.001
**HDL-Cholesterol (mg/dL)**	47.99 ± 14.35	47.88 ± 13.84	48.15 ± 14.97	0.821
**Total-Cholesterol (mg/dL)**	188.52 ± 48.23	189.74 ± 46.35	186.96 ± 50.49	<0.001
**BMI (kg/m** ^**2**^ **)**	32.26 ± 7.37	32.31 ± 7.37	32.18 ± 7.37	0.788
**NHHR**	3.23 ± 1.68	3.27 ± 1.67	3.19 ± 1.68	0.006

Notes: DKD, diabetic kidney disease; HDL, high-density lipoprotein; BMI, body mass index; PIR, poverty income ratio.

### Associations between NHHR and DKD

The relationship between NHHR and DKD was assessed using three models. Model 1, which included no adjustments, showed a negative correlation between NHHR and DKD prevalence. However, this relationship changed to a positive correlation after adjusting for variables. In Model 3, which included all adjustments, each one-unit increase in NHHR, was associated with a 22% increase in DKD prevalence (95% CI: 1.06, 1.40, *P* = 0.004). Stratifying NHHR into quartiles confirmed this positive association, with a significant trend observed (*P* < 0.001) shown in Table 2.

**Table 2:** The association between NHHR and DKD.

	Model1		Model2		Model3	
	OR [95% CI]	*P*	OR [95% CI]	*P*	OR [95% CI]	*P*
**NHHR**	0.97 (0.95, 1.00)	0.028	1.07 (1.04, 1.10)	<0.001	1.06 (1.03, 1.09)	<0.001
**Q1**	Ref	-	Ref	-	Ref	-
**Q2**	0.86 (0.76, 0.97)	0.015	0.95 (0.84, 1.08)	0.473	0.95 (0.83, 1.08)	0.448
**Q3**	0.90 (0.79, 1.01)	0.085	1.15 (1.01, 1.31)	0.031	1.15 (1.01, 1.31)	0.039
**Q4**	0.84 (0.75, 0.95)	0.006	1.27 (1.11, 1.45)	<0.001	1.22 (1.06, 1.40)	0.004
***P* for trend**	0.96 (0.92, 0.99)	0.021	1.09 (1.05, 1.13)	<0.001	1.08 (1.03, 1.12)	<0.001

Notes: Model 1, no adjustments. Model 2, adjusted for age, gender, and race. Model 3, adjusted for all presented covariates.

**Fig 2:**
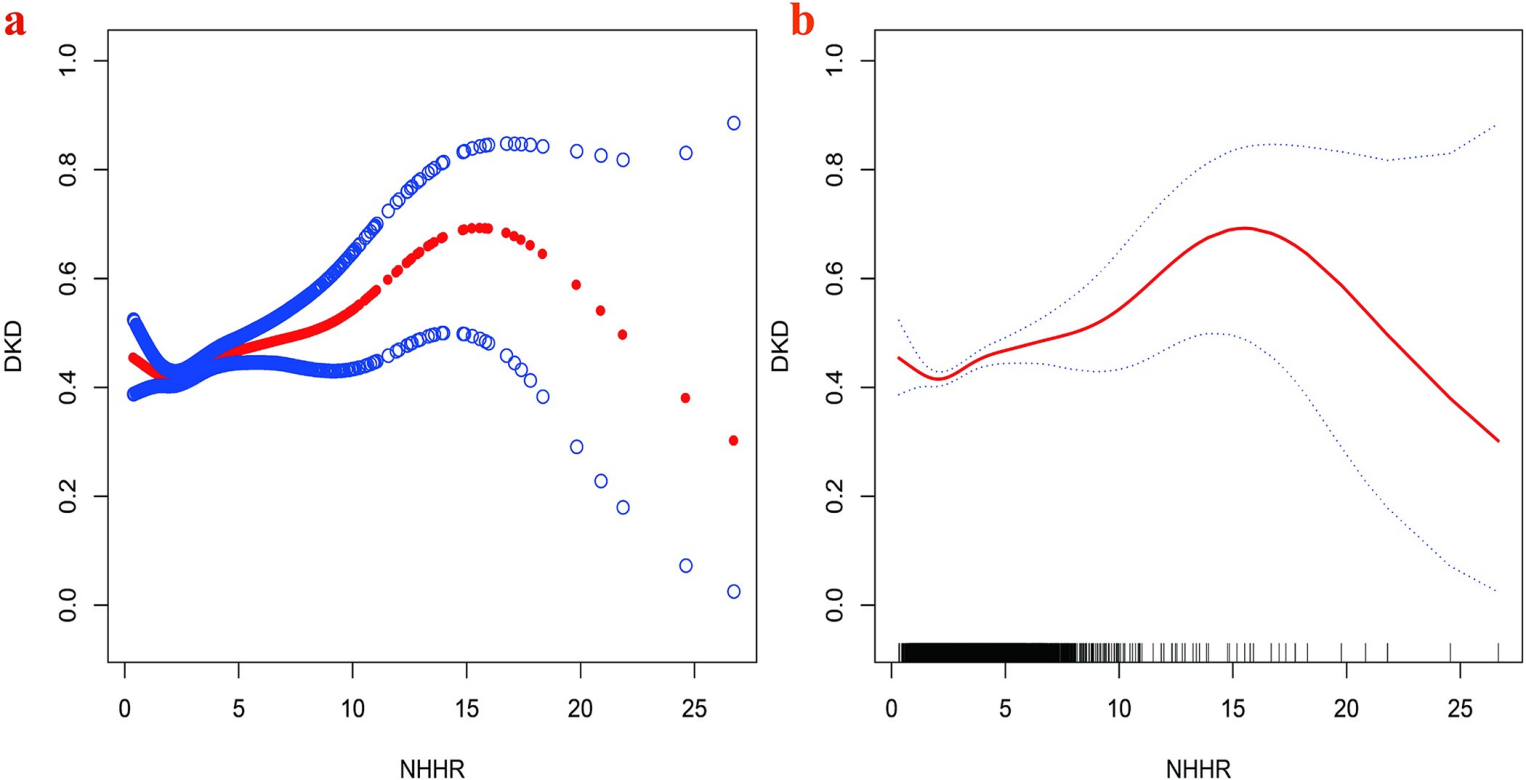
The association between NHHR and DKD. (a) The smooth curves fitting between variables. (b) The solid red line and the dashed blue line represent OR and 95%CI respectively. OR, Odds ratio (OR); CI, Confidence interval.

Smooth curve fitting analysis revealed a J-shaped relationship between NHHR and DKD prevalence (Fig 2). Threshold effect analysis identified an inflection point at 1.75, with an odds ratio (OR) of 1.07 (95% CI: 1.04, 1.10; *P* < 0.001), beyond this point, indicating that each unit increase in NHHR increased the risk of DKD by 7%. Before the inflection point, the OR was 0.77 (95% CI: 0.58, 1.02; *P* = 0.069), suggesting a less pronounced relationship (Table 3).

**Table 3:** Threshold effect analysis of NHHR on DKD.

Inflection point	Adjusted OR (95% CI)	*P*-value
**<1.75**	0.77 (0.58, 1.02)	0.069
**>1.75**	1.07 (1.04, 1.10)	<0.001
***P* for likelihood ratio test**	0.027	

### Subgroup analysis

**Fig 3:**
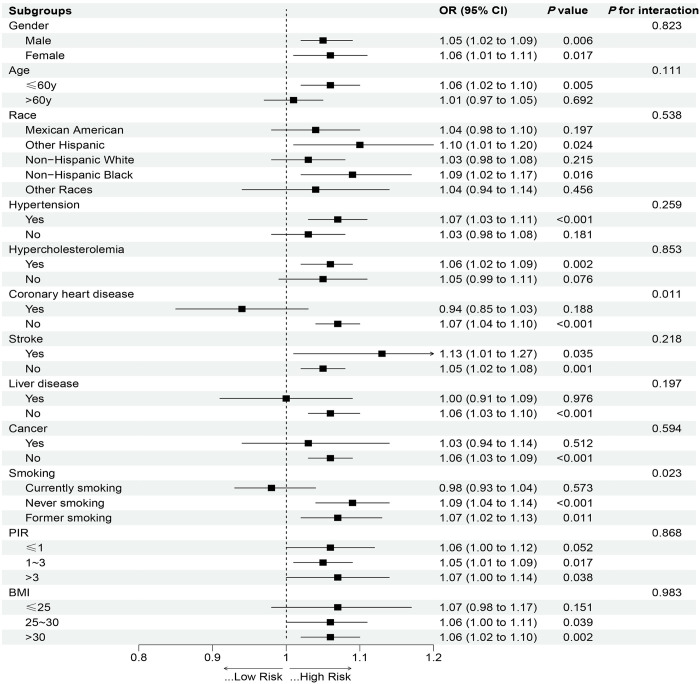
Subgroup analyses of the association between NHHR and DKD. BMI, body mass index; PIR, poverty income ratio.

Subgroup analyses and interaction tests were conducted to assess the robustness of findings across different demographic characteristics. Forest plots were employed to visualize the OR and 95% CI of the subgroups (Fig 3). The results indicated that the positive association between NHHR and DKD prevalence remained consistent across most subpopulations (*P* for interaction > 0.05). However, the association was notably stronger among individuals without coronary heart disease and those who did not currently smoke (*P* for interaction < 0.05).

## Discussion

Our findings support the initial hypothesis, revealing significant differences in NHHR between DKD and non-DKD populations. After adjusting confounders in three models, we observed a positive correlation between NHHR and DKD prevalence. Specifically, each one-unit increase in NHHR was associated with a 6% rise in DKD prevalence (95% CI: 1.03–1.09, *P* < 0.001). Threshold effect analysis identified an inflection point at 1.75, beyond which, each additional unit of NHHR was associated with a 7% increase in DKD prevalence. Subgroup analyses further confirmed the robustness of these results.

NHHR, as an emerging index for assessing atherosclerosis, has shown ambiguous with DKD. Lipid metabolism abnormalities have been a key focus in DKD research. Zhang et al. explored the role of lipid metabolism in early DKD stages by knocking out cholesterol efflux-related genes (ATP-binding cassette A1, ABCA1) in mice and exposing glomerular endothelial cells with ABCA1 deficiency to high glucose and cholesterol conditions. They found that ABCA1-deficient mice exhibited significant renal lipid deposition, inflammatory damage, and cell death. Similarly, ABCA1-deficient endothelial cells showed disruption of the endothelial glycocalyx barrier, cholesterol deposition, inflammation, and apoptosis. In contrast, ABCA1 overexpression protected these cells from high glucose and cholesterol-induced damage [28], suggesting that elevated cholesterol levels contribute to DKD progression. Consistent with our findings, a meta-analysis of 20 cohorts and 41,272 patients indicated that each 1 mmol/L increase in HDL-C was associated with a 22% increased DKD prevalence [29]. Another larger meta-analysis found that each 10 mg/dL increase in HDL-C levels was associated with a 6% decrease in DKD risk [30]. Notably, a large-scale, 4-year retrospective study demonstrated that patients with low HDL-C levels had worse renal outcomes compared to those with normal lipid levels. Specifically, each 10 mg/dL increase in HDL-C levels was associated with a 9% decrease in the risk of reduced eGFR and proteinuria [31], consistent with our findings.

However, the precise molecular mechanisms by which abnormal lipid metabolism contributes to DKD remain poorly understood. Substantial evidence indicates that dyslipidemia and iron-induced cell death due to oxidative stress are involved in DKD progression [32,33]. Moreover, abnormal lipid accumulation in podocytes and impaired autophagy are known to contribute to programmed cell death in DKD [34,35]. Importantly, dysregulation of lipid metabolism can exacerbate DKD progression by further disrupting lipid homeostasis [36,37]. Additionally, DKD-induced pathophysiological changes, such as alterations in the intestinal flora and increased intestinal barrier permeability, can negatively impact lipid metabolism and aggravate renal damage [38–40]. Key factors in DKD progression include disruptions in cholesterol synthesis, phagocytosis, exocytosis, and triglyceride metabolism, which are characterized by impaired fatty acid uptake and oxidation. Abnormalities in upstream genes can exacerbate kidney damage or alter kidney structure [41,42].

While much research has focused on HDL-C and its role in lipid-related diseases, less attention has been given to the impacts of TC and non-HDL-C. NHHR, as a novel index for assessing arterial atherosclerosis, accounts for the dual effects of non-HDL-C and HDL-C. It has been shown to offer superior predictive value and diagnostic efficacy compared to individual measures of HDL-C or non-HDL-C in evaluating lipid-related diseases. Our findings also reinforce the positive association between NHHR and the development of DKD.

Our study has several strengths, including the use of rigorous and reliable data, and a substantial sample size, which enhance the validity of our results. The construction of models and adjustments for potential confounders increase the credibility of our findings, and subgroup analyses further support the robustness of our results. However, there are limitations to consider: the cross-sectional design precludes establishing a causal relationship between NHHR and DKD; the complex sampling design may limit the generalizability of our findings; self-reported data from questionnaires may introduce bias; and the biennial data collection intervals may not capture rapid changes in disease states. Despite adjusting for major confounding factors, some residual confounding may still affect the results.

## Conclusion

Our findings indicate a positive association between NHHR and the prevalence of DKD. However, the underlying mechanisms remain inadequately explained. Future randomized controlled trials are essential to elucidate the causal relationship and provide further evidence for the early prediction and diagnosis of DKD.
